# Assessing neoadjuvant therapy recommendations in 19 national and international guidelines for rectal cancer

**DOI:** 10.1007/s10151-024-02969-5

**Published:** 2024-08-05

**Authors:** Pawel Mroczkowski, Selim Atay, Richard Viebahn

**Affiliations:** 1 Department for Surgery, Ruhr-University-Bochum, Knappschafts-University-Hospital, In der Schornau 23-25, 44892 Bochum, Germany; 2 Institute for Quality Assurance in Surgical Medicine Ltd., Otto-von-Guericke-University, Magdeburg, Germany; 3 Department for General and Colorectal Surgery, Medical University Lodz, Lodz, Poland

**Keywords:** Rectal cancer, Neoadjuvant therapy, Guidelines, Surgery

## Abstract

**Background:**

Treatment guidelines belong to the most authoritative sources of evidence-based medicine and are widely implemented by health-care providers. Rectal cancer with an annual incidence of over 730,000 new cases and nearly 340,000 deaths worldwide, remains a significant therapeutic challenge. The total mesorectal excision (TME) leads to a dramatic improvement of local control. The addition of neoadjuvant treatment has been proposed to offer further advancement. However, this addition results in significant functional impairment and a decline in the quality of life.

**Methods:**

This review critically assesses whether the recommendation for neoadjuvant treatment in current international guidelines is substantiated. A comprehensive search was conducted in July 2022 in PubMed resulting in 988 papers published in English between 2012 and 2022. After exclusions and proofs 19 documents remained for further analysis.

**Results:**

Of the 19 guidelines considered in this review, 11 do not recommend upfront surgery, and 12 do not address the issue of functional impairment following multimodal treatment. The recommendation for neoadjuvant therapy relies on outdated references, lacking differentiated strategies based on current utilisation of MRI staging; numerous guidelines recommend neoadjuvant treatment also to subgroups of patients, who may not need this therapy. Also statements regarding conflicts of interest are often not presented.

**Conclusions:**

An immediate and imperative step is warranted to align the recommendations with the latest available evidence, thereby affording rectal cancer patients a commensurate standard of care. A meticulous assessment of the guideline formulation process has the potential to avert heterogeneity in the future.

## Introduction

Guidelines play a pivotal role in the application of evidence-based medicine (EBM). They are widely accepted and typically undisputed. Improvement in rectal cancer therapy resulted from total mesorectal excision (TME) [1]. Further progress following the neo- and adjuvant strategies was suggested [2, 3], however, with significant impairment of the patients’ quality of life (QOL), resulting from the impaired functional outcome [4–7]. Current data derived from meticulous preoperative magnetic resonance imaging (MRI) underscore the post-surgical results without resorting to neoadjuvant regimens [8–14]. However, this perspective is often missed in guidelines that favour multimodal therapeutic options.

We undertook a thorough assessment of guidelines and consensus statements regarding the neoadjuvant treatment of rectal cancer over the past 10 years. We have checked the recommendations for this treatment and implementation of current evidence.

## Methods

**Fig. 1:**
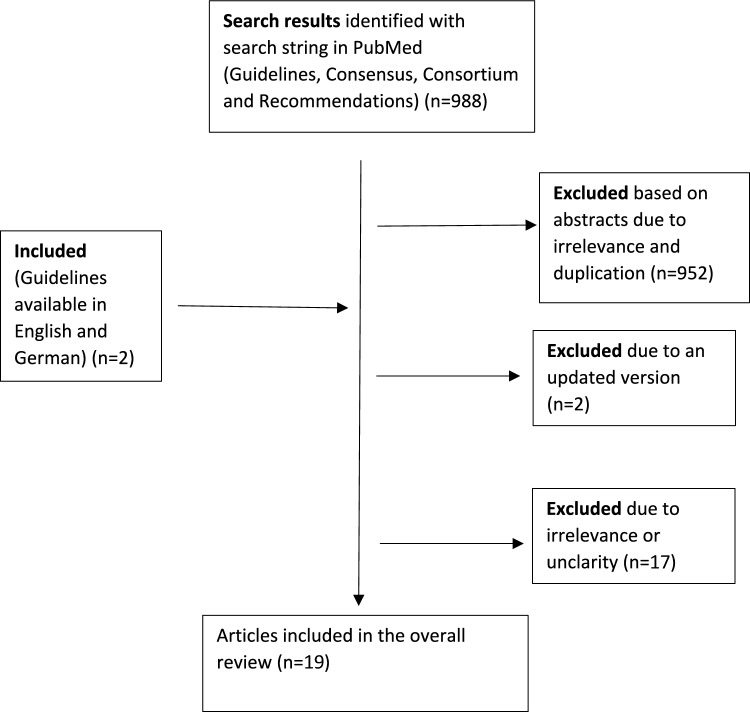
PRISMA diagram

To identify existing published guidelines and consensus documents a comprehensive search in PubMed was conducted in July 2022 using the following string:

The search was limited to papers published within the last 10 years, revealing 988 papers published in English between 2012 and 2022, with full-text accessibility. Based on abstracts, 952 were excluded, due to irrelevance or duplicity. Two more publications were accessible in German and English and added, while excluding two that became obsolete due to updates. Seventeen were excluded after evaluation of the full text. This resulted in 19 documents retained for further analysis (Fig. 1).

The aim of the search and selection process is the identification of documents, which can be regarded as a guideline, recommendation of a society, governmental body, etc. These documents can serve as instructions for care providers and sometimes also a legal aspect, being used as a benchmark in liability issues to determine whether a patient received standard care.

In this paper, all definitions and terms are used according to the meanings provided by a given guideline, as many values are not standardised. The aim is not to compare the guidelines themselves but to compare a given recommendation with existing and/or used evidence for that recommendation. Information about the respective authors’ conflicts of interest and surgical affiliations are mentioned as they could be possible co-founders affecting the observed discrepancies.

## Results

All documents described in this section do not mention functional impairment nor disclose conflicts of interest unless stated otherwise.

### Brazil

The 2021 guideline by the Brazilian Society of Surgical Oncology (BSSO) [15] recommends preoperative radio-chemotherapy based on fluoropyrimidine for stage II–III mid and low rectal adenocarcinomas. Moreover, the guideline omits neoadjuvant treatment for low-risk stage-II tumours, described as ‘T3a/b N0; with free mesorectal fascia; and amenable to sphincter preservation’. The rationale behind this sparing approach finds support from MERCURY [9], OCUM [12] and QUICKSILVER [13].

The recommendation for neoadjuvant treatment is substantiated by a collection of 17 references. None features a surgery-alone group.

### Canada

The Eastern Canadian Colorectal Cancer Consensus Conference (ECCCCC) in 2016 [16] and 2018 [17] (subsequent versions did not address indications) recommends preoperative radiation as the standard of care for cT3/T4N0 or any T-stage and lymph-node-positive disease. The sole reference cited is the NCCN guideline from 2014.

In the 2016 paper, 7 of the 34 authors declared conflicts of interest, whereas in the 2018 paper, 15 of 47 authors disclosed conflicts of interest.

### China

The 2020 National Health Commission of the People’s Republic of China guideline [18] advocates neoadjuvant chemoradiotherapy for T3 and/or N(+) tumours < 12 cm from the anus. They recommend upfront TME-surgery for a low-risk group meeting the following criteria: ≤ cT3a/b, cN0-2 (no cancer deposits), MRF(−), posterior wall localisation and EMVI(−). No sources are cited, and 23 of 90 authors have a surgical affiliation.

### England

The 2021 NG151 guideline of the National Institute for Health and Care Excellence (NICE) [19] recommends preoperative radiotherapy or chemoradiotherapy (CRT) for cT1–T2, cN1–N2, M0, cT3–T4, or any cN, M0 tumours. Supporting this recommendation is a 186-page review from 2020 [20].

NICE compared ‘any preoperative therapy versus no preoperative therapy’ among a list of 11 studies, with 5 of them including a surgery-alone group: Dutch-TME-Trial [2], Swedish Rectal Cancer Trial [3], MRC-CR 07 [21], ChiCTR-TRC-08000122 [22] and Zhang et al. [23]. The guideline mentions functional impairment and emotional/mental changes following therapy. The authorship is not delineated and the last update of committee members was in October 2017.

## ESMO

The 2017 European Society for Medical Oncology (ESMO) guideline [24] describes a risk-adapted treatment, recommending upfront TME when there is no indication on MRI that surgery will likely be associated with R2/R1 resection. ESMO reserves neoadjuvant therapy for threatened resection margin cases. The document stresses the role of high-quality surgery, requires auditing of local recurrence (LR) rates and addresses long-term functional impairment after combination therapy. The recommendation cites the analysis of surgical planes conducted in the MRC CR07 [25] and MERCURY trials [9]. Four of the seven authors report a conflict of interest and one has a surgical affiliation.

### France

The 2017 guideline by seven medical societies [26] recommends neoadjuvant therapy for T3/T4 tumours located in the middle/distal rectum and potentially for T2 of the anterior/distal rectum. No reference is directly linked to this recommendation. Four of ten authors disclose a conflict of interest, while no information regarding surgical affiliation is provided.

The 2016 guideline of the French Research Group of Rectal Cancer Surgery (GRECCAR) and the French National Society of Coloproctology (SNFCP) [27] offers the option of neoadjuvant therapy or immediate surgery for T1/T2 N(+) mid/low rectal cancer exhibiting sufficient circumferential resection margin (CRM) and T3 mid/low rectal cancer with a CRM > 2 mm. If a CRM ≤ 2 mm, neoadjuvant radio-chemotherapy is considered mandatory. The 2-mm limit was determined through analysis of the non-radiated cohort within the Dutch-TME trial [28]. The guideline underscores significantly poorer functional outcomes after neoadjuvant therapy. All authors have a surgical affiliation and declare no conflicts of interest.

### Germany

The 2017 German S3-Guideline was valid until 29 November 2022[29]^1^. At the time of proofreading (July 2024), an updated version has not yet been released. This guideline recommends chemoradiation or short-course radiation for cT3/4 and/or cN(+) low/mid rectal cancers. However, upfront surgery is recommended for cT1/2 mid/low tumours with questionable lymph-node involvement. Additionally, cT3a/b mid tumours with a minimal mesorectal fat infiltration (cT3a: < 1 mm, cT3b: 1–5 mm), are without suspected lymph-node involvement and without extramural vascular invasion (EMVI(−)).

The evidence-based recommendation cites eight references, of which only the Dutch TME-Trial [2] directly compares the neoadjuvant concept with TME-surgery alone. A total of 41 groups contributed, and the conflicts of interest disclosure is in Tables 23 and 24, pages 362–378[30].

### Great Britain and Ireland

The 2017 Association of Coloproctology of Great Britain and Ireland (ACPGBI) guideline [31] focus on the condition of the MRF. If the cancer is resectable and does not encompass this fascia, the standard recommendation is surgery alone. However, if MRI findings indicate a higher LR risk, e.g. T3c stage, mesorectal lymph-node involvement or EMVI, then preoperative radiotherapy may be considered.

The paper discusses early and late toxicities and functional impairment. Four authors report a conflict of interest.

### Greece

Through an online Delphi procedure engaging 100 experts across two voting rounds in 2016, a consensus was formulated by the Hellenic Society of Medical Oncologists (HeSMO) [32].

It recommends preoperative treatment for tumours categorised as ≥ cT3, CRM(+), EMVI and/or N(+) tumours and for T2 tumours situated in the lower rectum. The Dutch TME trial [2] and the MRC CR 07 trial [21] are the supporting evidence used. Within the text (not in the recommendations), a sentence mentions the potential for MRI-based selection of patients with T3a/b, who might not need preoperative treatment. This refers to a 2011 publication from the MERCURY study exploring the optimal cut-off distance for predicting CRM involvement [33].

Financial support by Sanofi Hellas for developing the consensus is disclosed.

### India

The Indian Council of Medical Research guideline, published in 2014 and online in 2021 [34], recommends short-course preoperative radiotherapy for T3 lesions and select T4 lesions demonstrating vaginal or peritoneal involvement and node-positive lesions, without compromised CRM. This recommendation cites a paper dealing with the surgical plane achieved in the MRC CR07/NCIC-CTG CO16 trials [25].

For node-positive T3/T4 lesions, where the CRM is threatened, the consensus advocating long-course neoadjuvant chemo-radiotherapy is supported by Sauer et al. [35]. A total of 4 of the 14 authors show affiliations to surgical institutions.

### Italy

In 2021, the Italian Society of Geriatric Surgery (SICG), Italian Society of Surgical Pathophysiology (SIFIPAC), Italian Society of Endoscopic Surgery and New Technologies (SICE) and the World Society of Emergency Surgery (WSES) published recommendations for multidisciplinary management of elderly (≥ 70 years) patients with rectal cancer [36].

The panel recommends short-course radiotherapy with delayed surgery for elderly patients with locally advanced stage II–III resectable cancer, advocating that neoadjuvant therapy offers ‘obvious advantages for local control comparing with surgery alone’. However, neither reference linked to this statement includes a surgery-alone group.

The document mentions functional impairment following neoadjuvant treatment. Only 1 of the 48 authors discloses a conflict of interest, while 32 have a surgical affiliation.

### Japan

The 2019 Japanese Society for Cancer of the Colon and Rectum (JSCCR) guideline [37] endorses preoperative radiotherapy for cT3/deeper or cN(+) tumours, based on sources from 1997 to 2003: a chapter in the Devita textbook, 2001 [38], the Swedish Rectal Cancer Trial, 1997 [3], a meta-analysis, 2000 [39] and a Colorectal Cancer Collaborative Group meta-analysis, 2001 [40].

The document mentions anal and bowel dysfunction after preoperative therapy.

The Guideline Evaluation Committee members disclosed connections to 39 corporations as conflicts of interest. Of the 33 institutions affiliated with the authors, 15 have a surgical character.

### Saudi Arabia

According to the 2014 Saudi Oncology Society guideline [41], patients diagnosed with T3–4 or N(+) lesions should undergo preoperative CRT. This directive is supported by the Swedish Rectal Cancer trial from 1997 [3].

A total of 2 of the 14 authors have a surgical affiliation.

### Spain

The 2016 Spanish Society of Medical Oncology (SEOM) guideline [42] describes neoadjuvant options without explicitly specifying the indication. All authors declare no conflicts of interest.

Conversely, the 2021 SEOM‑GEMCAD‑TTD guideline issued by the Multidisciplinary Spanish Group of Digestive Cancer and the Spanish Cooperative Group for Gastrointestinal Tumour Therapy [43] recommends neoadjuvant therapy for tumours in stage II or III. The only reference with a surgery-alone group is the Dutch-TME-Trial [2]. Four of the ten authors disclose conflicts of interest.

### USA

The 2020 guideline from the American Society of Colon and Rectal Surgeons (ASCRS) [44] recommends neoadjuvant therapy for stage II/III rectal cancer based on the 1997 Swedish Rectal Trial [3], the 2011 Dutch-TME trial [2] and the 2012 CAO/ARO/AIO-94 trial, [35]. The guideline acknowledges functional impairment and toxicity. All authors have a surgical affiliation.

In 2021, the American Society for Radiation Oncology (ASTRO) published an executive summary of its clinical practice guideline [45]. The authors express a strong recommendation for neoadjuvant radiotherapy in stage II–III rectal cancer, corroborated by the Swedish Rectal cancer Trial [3], NSABP R-03 [46], Sauer et al. [35], MRC CR 07 [21], Dutch-TME-Trial [2], Cochrane Review [47], Camma et al. [39] and Rahbari et al. [48]. However, for patients with stage II rectal cancer at lower risk of LR, neoadjuvant radiotherapy can be omitted. The authors define lower risk as a cT3a/b N0 tumour > 10 cm from the anal verge, mrCRM ≥ 2 mm and no mrEMVI. This statement lists four references: OCUM [12], MERCURY [10] and the Dutch-TME-trial [2, 49]. A total of 9 of 19 authors disclose conflicts of interests, while two have a surgical affiliation.

The initial search identified a 2018 version of the National Comprehensive Cancer Network (NCCN) Clinical Practice Guidelines rectal cancer [50]. On 21 September 2023, a new online-version (5.0) was published [51]. It recommends neoadjuvant treatment for most rectal cancers, with exceptions for T1–T2 N0 and T3 N0 low-risk, high rectal tumours. A total of six out of the 41 panel members have a surgical affiliation. Conflicts of interest are disclosed.

Table 1 summarises an overview of the main findings.

**Table 1:** Recommendations for neoadjuvant therapy, upfront surgery, functional impairment and conflicts of interest in analysed guidelines

Guideline	Neoadjuvant treatment criteria	Upfront surgery criteria	Functional impairment mentioned	Conflicts of interest disclosed
Brazil—BSSO	Mid and low adenocarcinoma (stages II and III)	T3a/b N0 with free mesorectal fascia	No	No
Canada ECCCCC 2016/2018	Standard/cT3/T4N0 or any T, lymph node positive	No	No	Yes
China—National Health Commission	T3 and/or N(+) < 12 cm from anus	≤ cT3a/b, cN0-2 (no cancer deposits), MRF(−), posterior wall, EMVI(−)	No	No
England—NICE	cT1–T2, cN1–N2, M0 or cT3–T4, any cN, M0	No	Yes	No
ESMO	Tumours with threatened resection margin	No R1/R2 resection in MRI predicted	Yes	Yes
France—intergroup	T4, T3 of the middle and distal rectum, potential indication for T2 of the anterior and distal rectum	No	No	Yes
France-GRECCAR/SNFCP	T3 mid/low rectal cancer with CRM ≤ 2 mm	T1/T2 N + mid/low rectal cancer with a sufficient CRM and T3 mid/low cancer with CRM > 2 mm	Yes	No
Germany	cT3/4 and/or cN(+) low and mid cancers	cT1/2 mid and low tumours with possible lymph node involvement and cT3a/b mid tumours with a minimal mesorectal fat involvement, without suspected lymph node involvement and EMVI(–)	Yes	Yes
Great Britain and Ireland	T3c stage, mesorectal lymph node involvement or EMVI	resectable rectal cancer not involving the mesorectal fascia	Yes	Yes
Greece	≥ cT3, CRM( +), EMVI and/or N( +) (recommendation) T2 tumours of the lower rectum (consideration)	MRI-based selection of patients in early III disease stage (T3a/b) based on CRM involvement	No	No^a^
India	T3, T4, N(+)	No	No	No
Italy	locally advanced stage II–III resectable rectal cancer	No	Yes	Yes
Japan	cT3 or deeper or cN(+)	No	Yes	Yes
Saudi Arabia	T3–4 or N(+)	No	No	No
Spain—SEOM	Not detailed	No	No	No
Spain—SEOM-GEMCAD-TTD	Stage II–III	No	No	Yes
USA—ASCRS	Stage II–III	No	Yes	No
USA—ASTRO	Stage II–III	cT3a/b N0 tumour > 10 cm from the anal verge, mrCRM ≥ 2 mm and no mrEMVI	No	Yes
USA—NCCN	all rectal cancer with exception of T1–T2 N0 and T3 N0 low risk, high rectal tumours	T1–T2 N0 and T3 N0 low risk, high rectal tumours	No	Yes

^a^Sanofi Hellas sponsored the consensus

## Discussion

Endorsement of neoadjuvant therapy in current guidelines appears to rely on a limited body of recent evidence, often with references that may not effectively support the statements.

### Historical evidence supporting the benefits of neoadjuvant therapy utilised in current guidelines

The Dutch-TME Trial (1996–1999) included 1861 patients who were randomised in a 1:1 ratio for TME preceded by 5 × 5 Gy versus TME alone (ratio 1:1). Notably, the 10-year LR was 5% and 11% (*p* < 0.0001), respectively [2].

This trial has raised two primary concerns. First, the number needed to treat (NNT), which is a metric that indicates the number of patients who must receive experimental treatment instead of the control treatment to prevent one additional patient from experiencing the study’s defined outcome. With 143 cases of LR (46 in the radiotherapy-surgery group and 97 in the surgery-alone group) within 10 years, the calculated NNT is 100.1. For every 100 patients treated, only one experiences a benefit, while the rest are exposed to side effects and complications.

Second, the staging protocol did not mandate dedicated MRI scans, which leaves the initial involvement of MRF unknown.

The papers authored by Sauer et al. [35, 52], reporting results of CAO/ARO/AIO-94 trial, do not inherently advocate neoadjuvant treatment due to their lack of a surgery-alone control group. These papers compare preoperative versus postoperative regimens and reported acute toxic effects of grade 3 or 4 in 27% of the patients in the preoperative group and 40% in the postoperative group, with long-term toxic effects in 14% and 24%, respectively.

The MRC CR07-NCIC-CTG C016 was a multicenter, randomised trial comparing preoperative radiotherapy versus selective postoperative CRT (12). The study recruited between 16 March 1998 and 5 August 2005 and published results in 2009 [21]. Following a median 4 year follow-up, the authors identified a significant 61% reduction in the relative risk of LR for patients who underwent preoperative radiotherapy, resulting in an absolute difference of 6.2% at the 3 year mark. The overall survival did not differ between the groups. However, these results are inapplicable today due to the trial’s initial staging protocol, which did not require a dedicated MRI, and thus the status of the MRF remains unknown.

The Cochrane review from 2007 [53], features a total of four applicable studies in its 2018 version [47]. Among them, only the Dutch trial, with the limitations discussed above, required the TME-technique. The three remaining trials include the Swedish RCT Trial (1987–1990) [3], MRC CR07/NCIC-CTG C016 trial (1998–2005) [21], and the Northwest Rectal Cancer Group trial (1982–1986) [54]. These trials included patients who were operated on without the TME technique, which would not be deemed adequate today.

A 2001 meta-analysis by the Colorectal Cancer Collaborative Group [40] included 22 randomised trials (19,631,984). Similarly, a 2000 meta-analysis of Camma et al. [39] included 14 trials (19,751,997), while the study of Rahbari et al. [48] considered 17 trials (1964–1999). Notably, only one mandated TME surgery. All these results therefore lack clinical consequences.

The Chinese ChiCTR-TRC-08000122 [22, 55] did not reveal any benefit of neoadjuvant therapy compared with surgery alone.

In 2008, Zhang et al. published in Academic Journal of Xi’an Jiaotong University results of a prospective randomised trial involving surgery combined with preoperative and postoperative radiotherapy [23]. The abstract indicated a study period of 1990–2002, while the text specified 1999–2002. The authors used Duke’s classification as inclusion criterion. The surgery-only group exhibited an LR of 64.3%, casting significant doubts in several aspects.

The NSABP R-03 trial [46] compared preoperative versus postoperative radiotherapy in patients treated in 1993–1999. The trial was terminated prematurely with 267 participants, (target: 900 patients). Furthermore, neither MRI nor TME were prerequisite for inclusion. Together with the lack of a surgery-only group, this study cannot be used as evidence advocating neoadjuvant therapy.

### Current evidence supporting MRI-based selective indication for neoadjuvant therapy omitted in numerous guidelines

The MERCURY-group presented their results in 2006 [8] and 2011 [9], and in 2014 [10], they published long-term results. The subcohort with a ‘good prognosis’ (MRI-predicted T2/T3a/T3b (< 5 mm spread from muscularis propria, regardless of MRI N stage), received neither preoperative nor postoperative radiotherapy and showed a 3% 5y-LR. The 2016 MERCURYII trial focused on low rectum cancers located ≤ 6 cm from the anal verge [11]. This trial showed a 1.6% pCRM-involvement in the group with no adverse MRI features, with a ‘safe’ low rectal cancer surgical resection plane (mrLRP), ≤ mrT3b, no extramural venous invasion (mrEMVI(−)), undergoing upfront sphincter-preserving surgery.

In 2018, the OCUM group released their findings advocating the avoidance of neoadjuvant CRT in low-risk patients [12], followed by final results in 2023 [14]. Patients presenting with cT2-4, any cN or cM0 rectal cancers were categorised based on the minimal distance between the tumour, suspicious lymph nodes or tumour deposits and the MRF. A subgroup demonstrating a distance > 1 mm underwent upfront TME, whereas those with a distance ≤ 1 mm and/or cT4 and cT3 tumours in the lower rectal third underwent nCRT followed by TME. The 5-year LR was 2.9% after upfront surgery and 5.7% following nCRT, while the 5-year rate of distant metastases was 15.9% and 30.5%, respectively.

The QUICKSILVER trial [13] assessed the feasibility of using MRI to identify patients eligible for primary surgery. The MRI-defined criteria for a favourable prognosis included: distance to MRF > 1 mm, definitive T2, T2/early T3, or definitive T3 with < 5 mm extramural depth of invasion, alongside the absence or equivocal extramural venous invasion. The positive CRM rate stood at 4.9%.

#### Further considerations

Modern imaging cannot reliably diagnose lymph-node involvement. A recent study that examined MRI features in 297 patients in stages IIA–IIIC [56] demonstrated that intraobserver agreement for lymph-node metastasis by the primary radiologist achieved a Cohen’s kappa (*k*) = 0.406, while multi-rater Fleiss kappa for interobserver agreement among four radiologists yielded *k* = 0.376 (0.175–0.577). The OCUM trial [57] indicated an MRI accuracy for lymph-node involvement of 56.5%, contrasting with an 86.5% accuracy for tumour-free CRM prediction and a negative predictive value of 98.1%. In Swedish population data, the sensitivity to detect N1–2 involvement is 42% [58]. With these results, lymph-node imaging remains insufficient for making decisions about treatment strategies, despite the fact that all mesorectal lymph nodes are excised during TME.

A number of the guidelines do not address functional impairment or toxicities. Considering that a multicentre analysis from 12 UK centres indicate only 7% of the patients with low rectal cancer, who underwent combined surgery and radiotherapy, reported normal bowel function, while 60% reported major impairment y[4], the omission of functional impairment should be scrutinised.

The limitations of the present paper consider primarily the heterogeneity of analysed guidelines and the difference in their roles in various countries and healthcare systems influencing the form and content of the presentation. Some of the documents are created by nationwide committees including many stakeholders, and some are products of individual scientific societies or a group of them. Furthermore, it is not explored in this paper how the individual countries deal with conflicting guidelines within their own borders or when national guidelines conflict with European guidelines. In addition, the volume of the documents varies from a few pages to several hundred pages. Despite all these nuances, the meaning of neglected current evidence and the danger of overtreatment remain the same.

Given the special role of guidelines within scientific literature, it is important to address their timely validity. Only the German guideline includes an expiration date, serving as a security mechanism to prevent the use of outdated statements. This requires the issuing body to maintain a continuous update process; otherwise, the guidelines’ relevance diminishes to merely reflect the standard of care at the time of its creation, sometimes several years old. Some issuing bodies regularly publish updates, providing an alternative solution to an expiration date.

## Conclusions

The prevalence of international guidelines advocating for neoadjuvant therapy in rectal cancer, despite the dearth of supporting evidence, raises concerns about the trust in evidence-based medicine. As a result, individuals with rectal cancer may be subjected to avoidable overtreatment, leading to unnecessary exposure to potential toxicities.

Addressing this issue is crucial. Collaborative efforts from the expert community are required to recalibrate the guidelines for rectal cancer, ensuring they closely align with rigorous evidence and prioritise patient well-being.

## Data Availability

All data in this review can be provided upon request.

## Footnotes

1 Guideline Registry in Germany, Colorectal Carcinoma Guidelines register no.: 021—007OL, *Arbeitsgemeinschaft der Wissenschaftlichen Medizinischen Fachgesellschaften (AWMF) e. V.*, at https://register.awmf.org/de/leitlinien/detail/021-007OL, accessed on 01 July 2024.
